# microRNAs and Inflammatory Immune Response in SARS-CoV-2 Infection: A Narrative Review

**DOI:** 10.3390/life12020288

**Published:** 2022-02-15

**Authors:** Beatrice Maranini, Giovanni Ciancio, Manuela Ferracin, Rosario Cultrera, Massimo Negrini, Silvia Sabbioni, Marcello Govoni

**Affiliations:** 1 Rheumatology Unit, Department of Medical Sciences, University of Ferrara, 44121 Ferrara, Italy; geppocia65@gmail.com (G.C.); gvl@unife.it (M.G.); 2 Department of Experimental, Diagnostic and Specialty Medicine (DIMES), University of Bologna, 40138 Bologna, Italy; manuela.ferracin@unibo.it; 3 Infectious Diseases, Department of Translational Medicine, University of Ferrara, 44121 Ferrara, Italy; rosario.cultrera@unife.it; 4 Laboratorio per le Tecnologie delle Terapie Avanzate (LTTA), Department of Translational Medicine, University of Ferrara, 44121 Ferrara, Italy; massimo.negrini@unife.it; 5 Department of Life Sciences and Biotechnologies, University of Ferrara, 44121 Ferrara, Italy; silvia.sabbioni@unife.it

**Keywords:** ncRNAs, miRNAs, lncRNAs, COVID-19, SARS-CoV-2, inflammatory response, immune response, cytokine storm

## Abstract

The current SARS-CoV-2 pandemic has emerged as an international challenge with strong medical and socioeconomic impact. The spectrum of clinical manifestations of SARS-CoV-2 is wide, covering asymptomatic or mild cases up to severe and life-threatening complications. Critical courses of SARS-CoV-2 infection are thought to be driven by the so-called “cytokine storm”, derived from an excessive immune response that induces the release of proinflammatory cytokines and chemokines. In recent years, non-coding RNAs (ncRNAs) emerged as potential diagnostic and therapeutic biomarkers in both inflammatory and infectious diseases. Therefore, the identification of SARS-CoV-2 miRNAs and host miRNAs is an important research topic, investigating the host–virus crosstalk in COVID-19 infection, trying to answer the pressing question of whether miRNA-based therapeutics can be employed to tackle SARS-CoV-2 complications. In this review, we aimed to directly address ncRNA role in SARS-CoV-2-immune system crosstalk upon COVID-19 infection, particularly focusing on inflammatory pathways and cytokine storm syndromes.

## 1. Introduction

SARS-CoV-2 worldwide outbreak in 2019 has become a global challenge. Old-age males, Hispanic and African American people, as well as patients with history of cardiovascular disease, hypertension, diabetes, obesity, malignancy and chronic diseases (such as kidney disease, arthritis and respiratory diseases) represent a vulnerable cohort of COVID-19 individuals [1].

The spectrum of clinical manifestations of SARS-CoV-2 is wide and ranges from asymptomatic or mild cases, characterized by fever and malaise, up to severe and complicated pictures, such as acute respiratory distress-like syndrome (ARD-LS), capillary leak syndrome, disseminated intravascular coagulation and multiorgan failure, leading to death in the most acute and serious cases [2]. Such critical courses of SARS-CoV-2 infection are thought to be driven by the so-called “cytokine storm” [3], an exaggerated response of the host immune system that induces the release of proinflammatory cytokines and chemokines, namely interleukin (IL)-1, IL-6 and tumor necrosis factor-a (TNF-a), as observed in macrophage activation syndrome (MAS) or secondary hemophagocytic lymphohistiocytosis (sHLH) [4,5]. Moreover, the hyperinflammatory state sustained by cytokine storm can also foster endothelial activation and dysfunction, and promote cell death, mainly by pyroptosis, resulting in severe systemic immune-mediated events [6]. Nonetheless, the primary mechanisms involved in the exaggerated inflammatory responses following SARS-CoV-2 infection remain to be fully elucidated.

In recent years, non-coding RNAs (ncRNAs) emerged as potential diagnostic and therapeutic biomarkers in both inflammatory and infectious diseases [7,8,9,10]. They do not codify proteins; instead, they produce RNAs effective in regulating gene expression and protein function. The two major classes of ncRNAs are microRNAs (miRNAs) and long ncRNAs (lncRNAs) [7]. miRNAs are crucial in post-transcriptional gene expression. Thus, they can control all biological processes, including inflammation, apoptosis and cell proliferation. A large number of studies exhibited the key role of miRNAs in the development of cardiovascular diseases, autoimmune diseases, neurodegenerative disorders and tumors [11,12,13]. The host immune system response against respiratory viruses is also associated with impaired expression of cellular miRNAs, which, consequently, can trigger antiviral and inflammatory pathways. Conversely, viral proteins can also escape antiviral immune responses through dysregulation of host cellular miRNAs [14]. The other class of ncRNA, lncRNAs, can also modulate gene expression and cellular processes, and may be involved in COVID-19 outcome [7]. Earliest studies supported the potential of miRNAs in modulating the amplitude of the innate immune response, significantly shaping inflammatory response; recent studies have also established their pivotal role in macrophage differentiation, infiltration and polarization [15]. Since the start and conclusion of inflammation are crucial to hamper tissue damage and uncontrolled progress of inflammatory reactions, a variety of mechanisms evolved in nature to modulate these processes, including negative and positive feedback loops [16].

Therefore, the identification of SARS-CoV-2 miRNAs and host miRNAs is an important research area to investigate the host–virus crosstalk in COVID-19 infection, in order to explain whether miRNA-based therapeutics can be employed to tackle SARS-CoV-2 complications [17,18].

In this narrative review, we analyze the latest literature data to list potential miRNAs driving immune response to SARS-CoV-2, with a particular focus on aberrant miRNA expression patterns of acute COVID-19 inflammatory response.

We searched Pubmed and Embase databases from pandemic inception to December 2021. COVID-19, SARS-CoV-2, non-coding RNAs, cytokine storm, inflammasome, inflammation and their respective MeSH terms were used as keywords. Specifically, we selected studies on miRNAs linked to inflammatory pathways or worse clinical outcomes. Only studies published in the English language were included, and the additional references quoted in these articles were also included. Both basic and clinical studies were selected.

## 2. Human miRNAs Involvement in Inflammatory Pathways Driven by SARS-CoV-2 Infection

Phylogenetically, SARS-CoV-2 is a positive-sense single-stranded RNA virus of the Coronavirus family with a genome size of approximately 30 Kb in length. It contains 16 non-structural and 4 structural proteins: Spike (S), Nucleocapsid (N), Membrane (M) and Envelope (E) [19]. SARS-CoV-2 shares high sequence identity with MERS-CoV, SARS-CoV and CoV-NL63, despite being more genetically similar to bat SARS-like coronaviruses [20,21]. SARS-CoV-2 genome sequences from 172 countries have been decoded and submitted to NCBI databases, providing a wide collection of information to aid the development of targeted antiviral therapeutics and vaccines [22]. Bioinformatics tools combined with molecular approaches are currently applied to genome characterization [23,24] in order to predict viral miRNAs and identify host’s miRNA-binding sites within the viral genome. Nevertheless, bioinformatics predictions must be experimentally validated to derive functional implications of miRNAs host–virus interaction [25].

It has been suggested that a central role for host miRNAs during viral infection lies in the modulation of cytokine response, either positive or negative, aiming at enhancing immune responses or suppressing potentially damaging ones [26]. 

As already widely established, immune hyperinflammatory response, mediated by dysregulated macrophages and innate and adaptive immunity, has been considered a common condition of severe COVID-19 cases [27]. Interleukins ensure essential function in the regulation of immune response, regulating proliferation, maturation, migration and adhesion of immune cells. The observed cytokine storm in SARS-CoV-2 infection, the uncontrolled release of proinflammatory mediators, such as TNF, IL-6, TNFα, IL-1β, IL-8, CCL2, CCL3, chemokine ligand CXCL10 and α-chain of IL-2 receptor, might be clinically associated with ARDS, diffuse alveolar damage, hypercoagulation and increased levels of acute phase reactants [28,29,30]. Other mediators of immune response, inflammation and apoptosis are NF-ĸB p65 (transcription factor p65) subunit [31] and the transforming growth factor beta (TGFB), whose signaling pathways are involved in induction of regulatory T cells, inhibition of B-cell and T-cell function, as well as favoring M2 rather than M1 macrophage responses [32]. Treatment with drugs inhibiting nuclear factor-kappa B (NFKB) activation proved to reduce lung inflammation in both SARS-CoV-infected cultured cells [33] and murine models, significantly increasing mice survival after SARS-CoV infection [34], while TGF-β signaling pathway proved to be targeted by miR-19a-3p and miR-19b-3p in a murine pulmonary fibrosis model [35].

Host miRNAs act as double-edged swords. As documented in previous studies, miRNAs can either enable viral immune evasion through targeting some pivotal host immune reactions [36], or decrease host responses to prevent acute tissue damage by targeting, for example, IGF1 or VEGF molecules [37,38]. As reported in studies performed before the pandemic, miR-146a and miR-155 are the first miRNAs enhanced by immune activation in immune cells; they modulate the Toll-Like Receptor (TLR)-signaling pathway, which triggers the production of a large variety of inflammatory cytokines, type I interferons (IFNs) and antiviral proteins through NFĸB pathway [39]. 

On this basis, more recently, Roganovic [40] proposed that the downregulation of circulating miR-146a observed in diabetes, obesity and hypertension may explain the more severe COVID-19 cases occurring in these patients. In another study, Roganovic [41] performed a computational analysis upon the interaction between miR-146 and -155 and genes encoding SARS-CoV-2 cellular entry facilitating factors. The study speculates that the upregulation of miR-146a and -155 in oral fluids of diabetic patients could promote ACE2 gene expression in oral tissues via negative regulation of ACE2 interactors. The authors point out, however, that upregulation of ACE2 expression in diabetes-associated periodontitis needs to be further validated in wider clinical studies. 

In recent decades, many studies, both in humans and animal models, have confirmed that miR-155 is consistently induced in different cell types (e.g., macrophages and T cells) during viral infections, playing a critical role in respiratory viral diseases throughout the modulation of antiviral responses and showing both a protective and/or a pathogenic role during viral respiratory infections. In a recent study, Arroyo et al. analyzed miR-155 levels in nasal mucosal samples from young children with viral respiratory infections not related to SARS-CoV-2. The study revealed how the secretion of miR-155 enhanced antiviral immunity and decreased respiratory disease severity in patients with high airway TH1 antiviral responses [42]. 

Additional studies expanded the involvement of a wide range of miRNAs in host immune responses in patients infected with SARS-CoV-2. In a study of Jafarinejad-Farsangi et al. [43], a few miRNAs, such as miR21, miR-16, let-7b, let-7e and miR-146a were predicted as targeting differentially expressed genes (DEGs) both in lung tissue and post-mortem specimens of patients infected with SARS-CoV-2. Among these genes were STAT1, CCND1, CXCL-10 and MAPKAPK2, all engaged in cytokine-involved signaling pathway. In a study of Centa et al. [44], the expression of miR-26a-5p, miR-29b-3p and miR-34a-5p proved to be significantly downregulated in lung biopsies of COVID-19 patients compared to healthy controls. Otherwise, miR-26a-5p expression was inversely correlated with IL-6 tissue expression, which is one of the cytokines directly regulated by this miRNA. In the same study, the reduced expression of miR-29b-3p corresponded with an increase in IL-4 and IL-8 levels in the COVID-19 group compared to controls. 

In a study by Li et al. [45], 14 peripheral blood samples, derived from COVID-19 patients (n = 10) and healthy donors (n = 4), have been collected to perform a whole-transcriptome sequencing. Lung involvement was considerable in the COVID-19 group, since ground-glass opacities were detected through chest CT images in 70% of individuals. Overall, 23 differentially expressed miRNAs and 410 lncRNAs were determined in the samples of the COVID-19 group compared to healthy controls. Gene ontology analysis showed that the upregulated mRNAs were mainly implicated in endogenous antigen-presentation mechanism, positive regulation of cytotoxic T lymphocytes and positive regulation of gamma–delta T-cell activation, while downregulated mRNAs were mainly affecting glycogen biosynthetic processes. These results suggested a potential pathogenic hypothesis for the aberrant cytokine production, as well as leukocyte differentiation and migration. The study also supports recent evidence indicating that lncRNAs may influence miRNA activity, highlighting the presence of an intricate endogenous RNA network between lncRNAs, mRNAs and miRNAs [46,47]. 

In Table 1, we summarized the main miRNAs discussed above. 

## 3. Human miRNAs–SARS-CoV-2 Virus Interaction 

Positive-strand RNA viruses mimic endogenous mRNAs and can thus be directly bound and regulated by host miRNAs [7]. In silico studies state that numerous human miRNAs can bind to SARS-CoV-2 RNA within its UTRs and protein-coding regions, but the functional implications of host miRNAs–virus interaction remain largely unknown [25]. Endogenous miRNAs can also target proteins essential for viral cell entry, such as angiotensin-converting enzyme 2 (ACE2), receptor (miR-200c) [54] or neural-entry cofactor neuropilin-1 (miR-24) [55]. 

A study of Guterres et al. [56] explored the miRbase database for human miRNAs that shared 100% identity of the 8mer seed region with SARS-CoV-2 genome regions, resulting in a perfect alignment of 11 nucleotides encompassing the seed region. The authors predicted 34 miRNAs for positive-sense viral RNA and 45 miRNAs for negative-sense viral RNA robustly binding to definite key SARS-CoV-2 genes. The identified miRNAs are also known to be involved in pulmonary and cardiac disorders and in the response to Influenza A viral infection, suggesting a contribution of miRNA deregulation to important clinical features of COVID 19. 

Several pathways might be deregulated by these host miRNAs to hamper cellular entry of SARS-CoV-2 virus to prevent the spread of the virions and curtail systemic symptoms. Among them, hsa-miR-17-5p and hsa-miR-20b-5p were found to be experimentally upregulated in H7N9 Influenza virus infection [57]. A recent study [58] predicted the existence of target motifs in the 5′UTR-Leader Sequence of SARS-CoV-2, attractive for non-canonical binding with endogenous human miRNAs, containing a “GGG” motif. The study identified 35 miRNA candidates, with a function involved in endothelial vascular injury, autophagy, lung fibrosis and inflammation, among others, which bind to the main sequences of the virus. Authors also identified 16 and 44 miRNA candidates capable of binding viral 3′UTR and the spike mRNA, respectively. A study of Nersisyan et al. [51] pointed out the possible interaction between miR-21-3p, miR-195-5p, miR-16-5p, miR-3065-5p, miR-424-5p and miR-421 and seven human coronavirus RNAs. To further verify this hypothesis, the authors analyzed datasets derived from mice lungs affected by SARS-CoV infection, revealing that the expression of miR-21-3p in mice lungs exhibits an 8-fold increase upon SARS-CoV infection. Computational predictions were also performed to compare the strength of miRNAs binding to different coronavirus UTRs, indicating a weaker binding in the novel SARS-CoV-2 virus compared with older, less pathogenic variants, thus subtending an evolutionary evasion from inhibition by endogenous miRNAs [59]. 

Another study suggested a possible sequestration of endogenous miRNAs by SARS-CoV-2 RNA, thus influencing the noncoding transcriptome of infected cells and creating a pro-viral environment, which needs to be experimentally validated [60].

In Table 2, we summarized the main miRNAs discussed above.

## 4. Viral miRNAs–Human Host Interaction

Viral miRNAs have been discovered in several human and non-human viruses, and SARS-CoV-2 is no exception. Here, we report the published evidence. 

Viral infection may raise the levels of cellular miRNAs, leading to upregulation of harmful antiviral molecules against viral replication. In addition, RNA viruses may interfere with endogenous miRNA, changing host transcriptome activities upon infection and halting the immune response to support favorable intercellular environment [63]. 

A study of Khan et al. [57] identified 122 and 106 host antiviral miRNAs against SARS-CoV and SARS-CoV-2, respectively. Among these, 27 miRNAs were found to target both viruses. In this study, the authors revealed that SARS-CoV-2 miRNAs can address different important organ-specific cellular functions and pathways. For instance, they showed that SARS-CoV-2-encoded miRNAs can target insulin pathway and genes associated with brain development, explaining the neurological clinical signs in COVID-19 infection, such as headaches, vomiting and nausea.

Rahaman et al. [64] applied a series of computational tools to predict 34 SARS-CoV-2-encoded miRNAs and their presumptive targets in human host; the immune and apoptotic pathways were selected as the most represented pathways (p53 signaling, cytokine receptor interaction, toll-like receptor (TLR) signaling, NOD-like receptor signaling and Jak-STAT signaling). A further investigation employing a dataset of SARS-CoV-2-infected cells revealed that 46 genes related to immune and apoptosis functions were deregulated during viral infection. In silico analysis confirmed that these downregulated genes were putative targets of 9 out of 34 predicted viral miRNAs. Moreover, 170 mature miRNAs from SARS-CoV-2 have been predicted as potentially targeting the host’s immune signaling pathways, as well as autophagy, IFN-I signaling, Wnt signaling, mTOR signaling and different pathways related to organ-specific functions, such as insulin signaling pathway and heart-development-related pathways, possibly mediating COVID 19-related complications in patients with comorbidities [57]. In a similar study [65], three precursor miRNAs were predicted. Their putative targets (FAM214A, PPM1E, NUFIP2 and FAT4) were found downregulated in SARS-CoV-2-infected A549 cells, suggesting the involvement of the predicted miRNAs in the fibrotic pathway. In another study [66], the authors performed a machine-learning-based miRNA prediction analysis for the SARS-CoV-2 genome to identify miRNA-like hairpins and predicted 29 potential precursor miRNAs. Among the predicted mature viral miRNA, 30 have been identified as targeting 1367 different human genes, exhibiting various molecular functions and pathways, principally involved in metabolic enzymatic interconversion (110 genes) and in host transcriptional processes (96 genes). Based on the potential targets of the host transcription machinery, SARS-CoV-2 may obviate RNA polymerase II to assemble promoters of host genes at the initiation step and provide opportunities for viral mRNA to elude degradation. Satyam et al. [67] developed a systematic pipeline to computationally predict putative viral mature miRNAs and their targeted transcripts. They predicted six putative miRNAs that target immune-related genes and, majorly, genes involved in cell proliferation/differentiation/signaling and senescence. A network analysis of their results allowed the authors to conclude that the predicted SARS-CoV-2 miRNAs could directly or indirectly target host genes involved in the “Cytokine Storm Pathway” linked to severe COVID-19. 

Merino et al. [68] implemented deep learning and discovered 12 candidate miRNA precursors in the viral protein-coding genome and confirmed their prediction on a small RNA-seq dataset from SARS-CoV-2-infected human cells. They suggested that the expression of eight mature miRNA-like sequences may modulate the host transcriptome upon infection; moreover, they indicated as possible targets of SARS-CoV-2 predicted miRNAs, among others, genes linked to the SARS-CoV-2 infection, including ACE2, CXCL3, JAK2 and STAT2. The authors also compared the SARS-CoV-2 pre-miRNA sequences with those from bat and pangolin species and proposed a novel miRNA-like sequence acquisition, advocating their involvement in inter-species boundaries jump. 

## 5. Therapeutic Potential of miRNAs-Based Treatments in COVID-19

microRNA-based therapeutics represent a potentially relevant area of investigation, since miRNAs targeted inhibition could mediate a direct antiviral effect and/or counteract the inflammatory response during infection [69]. In a review of Abedi et al. [70], several miRNAs involved in the inflammatory response to SARS-CoV-2 infection are suggested as potentially useful against the inflammatory cascade, in cytokine storm attenuation and in acute lung injury reduction. Rohani et al. [71] explored the CLASH database, which contains validated interactions between human miRNAs and human mRNAs, to identify host miRNAs with a therapeutic potential, that may have efficient interactions with SARS-CoV-2 unstructured conserved regions (UCRs) and a low affinity to human mRNAs. A total of seven candidate miRNAs (hsa-miR-374a-5p, hsa-miR-548b-3p, hsa-miR-1-3p, hsa-miR-224-5p, hsa-miR-98-5p, hsa-miR-26a-2-3p, hsa-miR-192-3p) potentially targeting several SARS-CoV-2 UCRs, could facilitate antiviral IFN-β immune response, prevention of cell autophagy and degradation of viral components. These miRNAs preferentially bind viral UCRs, rather than targeting host mRNAs, and were proposed as possible therapeutic molecules for COVID-19 intervention. 

With the same aim, the authors also identified bat-specific miRNAs potentially able to target SARS-CoV-2 UCRs, in particular nsp3, nsp4 and nsp6, which are involved in the replication/transcription complex (RTC) and the rearrangements of host-derived membranes in organelle-like replicative structures. The bat-specific miRNAs and others candidate miRNAs derived from 286 other species showed relevant affinities in binding with the spike protein and were computationally screened to predict potential interference with host biological processes.

Since exosomes secreted by mesenchymal stem cells can modulate the immune system, reducing cytokines that cause cytokine storms, miRNA-loaded exosomes have recently been suggested as an experimental therapeutic option [53,72]. Through bioinformatics analysis, Schulz et al. predicted miRNAs from different stem cell tissue sources, some of which may contemporarily decrease inflammatory agents, regulate cell death genes, as well as coagulation factors, consequently preventing tissue damage and coagulation disturbances [53,72]. Nevertheless, concerns regarding safety and efficacy of the therapeutic potential of this approach need to be addressed [73].

On the basis of the examples related to other viruses, such as EBV-miR-BART10-5p in Epstein–Barr virus [74] or miR-122 in hepatitis C treatment [75], relevant viral or host miRNAs could be targeted by therapeutic antagomirs [66]. A study of Kumar-Soni et al. [76] focused on examining the effect of anti-miR-155 on the lungs of SARS-CoV-2-infected mice, which expressed human ACE2 (hACE2-transgenic mice). The authors reported that anti-miR-155-treated hACE2-transgenic mice infected with SARS-CoV-2 exhibit reduced levels of proinflammatory cytokines, and equally importantly, they have increased anti-inflammatory cytokine responses in lungs. These results suggest that anti-miR-155 could represent a novel therapeutic target to address the lung cytokine storm induced by SARS-CoV-2 infection. This finding is in line with Chow et al. [77] and with Emanuel et al. [78], who demonstrated in vitro an increase in miR-155 expression in SARS-CoV-2-infected epithelial cells and in a non-small-cell lung cancer cell line, respectively.

Successful strategies have emerged to improve miRNA’s safety and potential use as vaccine candidates [79]. In recent years, miRNAs have been used to develop live attenuated vaccines, such as the poliovirus vaccine. In addition, miRNA-containing vaccines do not replicate in neuronal cells but may replicate in the gastrointestinal tract, where they act as an immunogen [80]. For all these reasons, miRNAs are considered attractive candidates for the development of vaccines and antivirals, even if further research would allow a better understanding of the role of vaccines in miRNA regulation [81].

In Table 3, we summarized the main miRNAs discussed above.

## 6. Human miRNAs as Biomarkers of Severe COVID-19

Host miRNAs linked to worse prognosis. Many miRNAs have been predicted to be linked to viral pathogenicity and host responses. However, it remains to be elucidated which ones may be linked with worse prognosis or the need for Intensive Care Unit (ICU) admission. A study of Arisan et al. [82] identified seven key miRNAs (miRs 8066, 5197, 3611, 3934-3p, 1307-3p, 3691-3p, 1468-5p) mainly involved in host response to virus. Among them, miR-1307 is a lung-tissue-associated miRNA, involved in transforming growth factor (TGF)-β and semaphoring signaling, as well as inflammatory responses. TGF-β has been previously reported to be a particularly important target in lung development in newborns [83], and it has been associated with a more severe grade of pulmonary hypertension in systemic sclerosis [84]. The most challenging issues with SARS-CoV-2-infected patients are oxygen therapy and the need for mechanical ventilation, all aspects that may correlate with the involvement of miR-1307-3p [85]. Concerning patients who need advanced care, a study of Gonzalo-Calvo et al. [48] demonstrated in blood samples that miR-16-5p, miR-92a-3p, miR-98-5p, miR-132- 3p, miR-192-5p and miR-323a-3p showed significant suppression in patients who did not survive the ICU admission. Moreover, in the same study of Arisan et al., not only miR-1307-3p, but also miR-8066, miR-5197-3p and miR-3691-3p were associated with TGF-β pathway, cytokine cascade and oxidative stress, all well-known hallmarks of pulmonary damage.

A study by Tang et al. [46] analyzed the global miRNA and transcriptome expression profile in PBMC isolated from moderate or severe COVID-19 patients. The results indicated that miR-146a-5p and miR-21-5p play opposite but fundamental roles during SARS-CoV-2 infection. Their observed downregulation in severe COVID-19 cases influences the production of IRAK1 (interleukin-1 receptor-associated kinase), IRAK2 and TRAF6 (tumor necrosis factor receptor-associated factor 6) in immune cells. The authors identified a complex regulatory network, involving miRNAs, long ncRNAs and mRNAs that were dysregulated in server COVID-19 patients. These alterations contribute to the hyperactivation of the immune response and hyperinflammation. Moreover, miR-21-5p also directly target CCL20 (C-C motif chemokine ligand 20) and MYC (a family of regulator genes and proto-oncogenes), whose overexpression fosters T-cell activation. Therefore, the strong correlation between these two miRNAs and disease severity indicates that miR-146a-5p and miR-21-5p might be key regulators of COVID-19 pathogenesis and host immune response. In the same work, circulating miR-146a-5p, miR-21-5p and miR-142-3p were also identified as potential biomarkers of disease deterioration and COVID-19 severity, and possibly also as candidate therapeutic targets.

miRNAs as circulating biomarkers. The potential usefulness of circulating miRNAs as disease biomarkers has been widely investigated in several pathologic conditions. In the COVID-19 setting [86], the analysis of whole blood samples collected from 46 COVID-19 patients who recovered from moderate (MM) or severe (SC) disease and 24 healthy subjects revealed that miR-155 and miR-130a levels were remarkably higher in the MM group compared to the SC group.

Despite it being conceivable that the expression pattern of miRNAs related to inflammation would differ during the acute phase and the post-acute phase of the infection, a study of Donyavi et al. [52] identified miR-29a-5p, 146a-3p, 155-5p and let-7b-3p in peripheral blood mononuclear cell (PBMC) of COVID-19 virus-infected patients both in the acute and post-acute phases of the disease. 

Other studies highlighted the possible value of circulating cell-free miRNAs, instead of blood-cell-associated miRNAs, as biomarkers for disease diagnosis/prognosis. A study compared the signature of circulating miRNAs in plasma of COVID-19 patients versus healthy donors [61]. The authors identified two downregulated (miR-17-5p and miR-142-5p) and six upregulated (miR-15a-5p, miR-19a-3p, miR-19b-3p, miR-23a-3p, miR-92a-3p and miR-320a) miRNAs in SARS-CoV-2-infected patients. The results of the ROC curve analyses indicate that plasma miR-19a-3p, miR-19b-3p and miR-92a-3p expression levels may be relevant as potential diagnostic biomarkers. Moreover, the authors suggest that upregulated levels of miR-19a/b observed in plasma of SARS-CoV-2-infected patients versus healthy individuals could be involved in the inflammatory storm seen in COVID-19 via inhibition of the immunosuppressive and anti-inflammatory role ensured by TGF-β signaling pathway. Interestingly, another study by Sabbatinelli et al. [87], carried out in a cohort of 30 patients with multifocal interstitial pneumonia due to SARS-CoV-2 infection, showed that COVID-19 patients who did not respond to anti-IL-6 therapy (Tocilizumab) had lower serum levels of miR-146a-5p and experienced the most adverse outcome during the follow-up. Roganovic et al. [40] speculated that patients with diabetes, obesity and hypertension, due to downregulation of circulating miRNA-146a, may be prone to severe COVID-19.

In a study aimed at identifying potential biomarkers of disease severity, altered levels of circulating inflammatory miRNAs were investigated in severe COVID-19 patients requiring invasive ventilation and compared to healthy controls and influenza–ARDS patients [49]. Fibrosis-associated miR-21 was found to be overexpressed in acute COVID-19 patients compared with healthy and ARDS controls, and ROC curve analyses indicate that serum levels of miR-155, miR-208a and miR-499 are clearly distinct between COVID-19 and influenza–ARDS patients. Moreover, the upregulation of miR-21, miR-155, miR-208a and miR-499 in COVID-19 recovered patients correlated with the probability of chronic myocardial damage and inflammation, raising awareness on appropriate management and monitoring. 

Finally, circulating miRNAs may also affect regulatory networks associated with cytokine storms in COVID-19 patients. In a study of Yang et al. [88], plasma-cell-free circulating RNAs (cfRNAs) profiles of COVID-19 patients showed, among others, lower levels of miR-451a when compared with healthy donors and a significantly higher mRNA expression of the IL-6R target. miR-451a was shown to be one of the top five downregulated microRNA in this cohort of COVID-19 patients. The authors also identified three upregulated lncRNAs (LOC105371414, LOC105374981 and LOC107987081), carrying miR-451a binding sites, possibly competing with IL-6R for miR-451a binding. Lower levels of miR-374a were also detected in COVID-19 patients with respect to healthy controls, while its target, CCL2, is involved in acute respiratory distress syndrome (ARDS) and cytokine storms in COVID-19 patients. The authors thus suggested that both miRNAs, along with enhanced lncRNA levels, may promote IL-6R/CCL2 translation in COVID-19 patients, contributing to IL-6-induced cytokine storms.

In Table 4, we summarized the main miRNAs discussed above.

## 7. Conclusions

In this review, we aimed to investigate miRNA crosstalk between SARS-CoV-2 and the host system, with a focus on inflammatory pathways and cytokine storm syndrome, mechanisms wherein lie the difference between mild and severe COVID-19 cases. 

Several studies described deregulation of microRNAs in tissues and in specific cellular populations infected by SARS-CoV-2, highlighting miRNA involvement in inflammation, immune response and cytokine storm. However, the exact role in disease pathogenesis is not fully elucidated [66]. 

Based on studies of miRNA expression, even during other viral infections [36] it is conceivable that the antiviral response of cells after SARS-CoV-2 infection may include the production of miRNAs directly or indirectly targeting viral processes. On the other hand, the virus may potentially produce viral miRNAs in order to escape from the host defense system.

However, to date, there have been conflicting results in the literature; for example, sometimes the same miRNA appears to be up- or downregulated in divergent ways, according to different studies. Not all miRNAs targeted genes have been established, nor has their complex crosstalk. Moreover, this review confirms that the identification of the potential host–virus miRNA interactions, mainly based on computational prediction analysis, needs further experimental verification.

Nevertheless, two important implications of miRNA deregulation in COVID-19 patients emerge: the potential therapeutic and diagnostic/prognostic use of miRNAs. Circulating miRNAs in patient serum could be used to monitor clinical progression of COVID-19 and the outcome [56]. Regarding the therapeutic potential, mimics or antagomiR molecules, their use could be envisaged for restoring a level of antiviral miRNAs or inhibiting the level of proinflammatory ones, only once the specific interactions are assessed, and any potential harmful off-target effect is excluded [7]. An example of miRNA-based antiviral treatment is the approved Miravirsen drug, based on the modified anti-miR-122 molecule to counteract the replication of the Hepatitis C virus sustained by miR-122 [90]. 

Further studies are therefore needed to fully validate the relationship between miRNAs and other ncRNAs and severe inflammatory response in SARS-CoV-2 infection, with the final goal to identify biomarkers for clinical assessment and, hopefully, novel therapeutic targets. 

## Figures and Tables

**Table 1:** Summary of principal human miRNAs involved in inflammatory pathways driven by SARS-CoV-2 infection.

miRNA	Up/Down-Regulation	Principal Target Genes	Function	Ref.
miR-16-5p	Downregulation	NA	NA	[43,48]
miR-21	Upregulation	NA	NA	[43,46,49,50,51]
miR-26a-5p	Downregulation	IL-6	Stimulating acute phase protein synthesis, as well as the production of neutrophils in the bone marrow. It supports the growth of B cells and is antagonistic to regulatory T cells.	[44]
miR-29b-3p	Downregulation	IL-4	Stimulation of activated B-cell and T-cell proliferation and the differentiation of B cells into plasma cells. It is a key regulator in humoral and adaptive immunity. IL-4 induces B-cell class switching to IgE and upregulates MHC class II production.	[44]
		IL-8	Neutrophil chemotactic factor, which has two primary functions. It induces chemotaxis in target cells.	[44]
miR-34a-5p	Downregulation	NA	NA	[44]
		STAT3	Induces the expression of many cytokines, chemokines and other mediators, such as interleukin-6 and cyclooxygenase 2.	[52]
		IL-1b	Induces prostaglandin synthesis, neutrophil influx and activation, T-cell activation and cytokine production, B-cell activation and antibody production and fibroblast proliferation and collagen production. Promotes Th17 differentiation of T cells.	[52]
		TLR2	The protein encoded by this gene is a member of the toll-like receptor (TLR) family, which plays a fundamental role in pathogen recognition and activation of innate immunity.	[52]
miR-146	Upregulation	CLEC5A	By suppression of CLEC5A/TLR2 signaling- inhibition of production of cytokines (TNF-α, IL-1, IL-6, IL-8, IL-17) and chemokines. Overactivation of CLEC5A/TLR2 is detrimental during acute viral infections.	[41]
		KRAS	Inhibition of Ras/NFkB signaling; reduction in production of proinflammatory cytokines: IL-17, IL-22, IFN-γ, TNF-α, IL-6.	[41]
		NRAS	Reduction in production of proinflammatory cytokines: IL-17, IL-22, IFN-γ, TNF-α, IL-6.	[41]
		CAT	Oxidative stress	[41]
		STAT1	Mediates cellular responses to interferons (IFNs), cytokine KITLG/SCF and other cytokines and other growth factors.	[43]
		STAT3	Induces the expression of many cytokines, chemokines and other mediators, such as interleukin-6 and cyclooxygenase 2.	[52]
		IL-1b	Induces prostaglandin synthesis, neutrophil influx and activation, T-cell activation and cytokine production, B-cell activation and antibody production and fibroblast proliferation and collagen production. Promotes Th17 differentiation of T cells.	[52]
		TLR2	The protein encoded by this gene is a member of the toll-like receptor (TLR) family, which plays a fundamental role in pathogen recognition and activation of innate immunity.	[52]
miR-155	Upregulation	KRAS	Inhibition of Ras/NFkB signaling; reduction in production of proinflammatory cytokines: IL-17, IL-22, IFN-γ, TNF-α, IL-6.	[41]
		CD33	Upregulation of proinflammatory cytokines: IL-1β, IL-8, TNF-α.	[41]
		TGFB1	Regulation of various cell activities inside the cell, including the growth and division (proliferation) of cells, the maturation of cells to carry out specific functions (differentiation), cell movement (motility) and controlled cell death (apoptosis).	[52]
		FOXP3	Maintaining homeostasis of the immune system by allowing the acquisition of full suppressive function and stability of the Treg lineage and by directly modulating the expansion and function of conventional T cells.	[52]
		NA	NA	[49]
miR-Let-7b-3p	Upregulation	TNF	Potent pyrogen causing fever by direct action or by stimulation of interleukin-1 secretion; it is implicated in the induction of cachexia. Under certain conditions, it can stimulate cell proliferation and induce cell differentiation. Impairs regulatory T-cell (Treg) function.	[43,52]
		TGFB	Lung fibrosis, fluid homeostasis; potent chemokine-like molecule, neutrophil recruitment.	[52]
		NFKB1	NF-kappa-B is a pleiotropic transcription factor present in almost all cell types, and it is the endpoint of a series of signal transduction events that are initiated by a vast array of stimuli related to many biological processes, such as inflammation, immunity, differentiation, cell growth, tumorigenesis and apoptosis.	[52]
miR-Let-7e-5p	NA	RIPK1	Controls multiple signaling pathways leading to inflammation and apoptotic or necroptotic cell death.	[43,53]
		CASP8	Encodes a member of the cysteine–aspartic acid protease (caspase) family. Sequential activation of caspases plays a central role in the execution phase of cell apoptosis.	[53]
		TNF	Potent pyrogen causing fever by direct action or by stimulation of interleukin-1 secretion, and it is implicated in the induction of cachexia. Under certain conditions, it can stimulate cell proliferation and induce cell differentiation. Impairs regulatory T-cell (Treg) function.	[43,53]

Legend: NA = not available.

**Table 2:** Summary of principal human miRNAs involved in SARS-CoV-2 virus–host interaction.

miRNA	Up/Down-Regulation	Principal Target Genes	Function	Ref.
miR-17-5p	Downregulation	NA	NA	[61]
	Downregulation	MYC	Controls cell cycle, cell growth, apoptosis, cellular metabolism and biosynthesis, adhesion and mitochondrial biogenesis.	[62]
		IL-6	Stimulating acute phase protein synthesis, as well as the production of neutrophils in the bone marrow. It supports the growth of B cells and is antagonistic to regulatory T cells.	
		ICAM	Roles in cell proliferation, differentiation, motility, trafficking, apoptosis and tissue architecture.	
		VEGFA	Induces endothelial cell proliferation, promotes cell migration, inhibits apoptosis and induces permeabilization of blood vessels.	

Legend: NA = not available.

**Table 3:** Main potential therapeutic targets in SARS-CoV-2 infection.

miRNA	Up/Down-Regulation	Principal Target Genes	Function	Ref.
miR-26a-5p	Downregulation	IL-6	Stimulating acute phase protein synthesis, as well as the production of neutrophils in the bone marrow. It supports the growth of B cells and is antagonistic to regulatory T cells.	[44]
miR-98-5p	Downregulation	NA	NA	[48]
miR-192-5p	Downregulation	NA	NA	[48]
miR-374a	Upregulation	MYC	Controls cell cycle, cell growth, apoptosis, cellular metabolism and biosynthesis, adhesion and mitochondrial biogenesis.	[62]
		IL-6	Stimulating acute phase protein synthesis, as well as the production of neutrophils in the bone marrow. It supports the growth of B cells and is antagonistic to regulatory T cells.	
		ICAM	Roles in cell proliferation, differentiation, motility, trafficking, apoptosis and tissue architecture.	
		VEGFA	Induces endothelial cell proliferation, promotes cell migration, inhibits apoptosis and induces permeabilization of blood vessels.	

Legend: NA = not available.

**Table 4:** Main potential miRNAs acting as biomarkers of severe SARS-CoV-2 infection.

miRNA	Up/Down-Regulation	Principal Target Genes	Function	Ref.
miR-15a-5p	Upregulation	NA	NA	[61,89]
miR-17-5p	Downregulation	NA	NA	[61]
	Downregulation	MYC	Controls cell cycle, cell growth, apoptosis, cellular metabolism and biosynthesis, adhesion and mitochondrial biogenesis.	[62]
		IL-6	Stimulating acute phase protein synthesis, as well as the production of neutrophils in the bone marrow. It supports the growth of B cells and is antagonistic to regulatory T cells.	
		ICAM	Roles in cell proliferation, differentiation, motility, trafficking, apoptosis and tissue architecture.	
		VEGFA	Induces endothelial cell proliferation, promotes cell migration, inhibits apoptosis and induces permeabilization of blood vessels.	
miR-19a-3p	Upregulation	TGFb	Lung fibrosis, fluid homeostasis; potent chemokine-like molecule, neutrophil recruitment.	[61]
miR-23a-3p	Upregulation	NA	NA	[61]
miR-29a-5p	Upregulation	IL-4	Stimulation of activated B-cell and T-cell proliferation and the differentiation of B cells into plasma cells. It is a key regulator in humoral and adaptive immunity. IL-4 induces B-cell class switching to IgE and upregulates MHC class II production.	[52]
miR-29b-3p	Downregulation	IL-4	Stimulation of activated B-cell and T-cell proliferation and the differentiation of B cells into plasma cells. It is a key regulator in humoral and adaptive immunity. IL-4 induces B-cell class switching to IgE and upregulates MHC class II production.	[44]
miR-208a	Upregulation	IL-8	Neutrophil chemotactic factor, which has two primary functions. It induces chemotaxis in target cells.	[44]
miR-320a	Upregulation	NA	NA	[61]
miR-323a-3p	Downregulation	NA	NA	[48]
miR-374a	Downregulation	NA	NA	[88]
miR-451a	Downregulation	IL-6	Stimulating acute phase protein synthesis, as well as the production of neutrophils in the bone marrow. It supports the growth of B cells and is antagonistic to regulatory T cells.	[88]
miR-499	Upregulation	NA	NA	[49]
miR-1307	Upregulation	TGFB	Lung fibrosis, fluid homeostasis; potent chemokine-like molecule, neutrophil recruitment.	[82]
miR-3691-3p	NA	TGFb	Regulates various cell activities inside the cell, including the growth and division (proliferation) of cells, the maturation of cells to carry out specific functions (differentiation), cell movement (motility) and controlled cell death (apoptosis).	[82]
miR-5197-3p	NA	TGFb	Regulates various cell activities inside the cell, including the growth and division (proliferation) of cells, the maturation of cells to carry out specific functions (differentiation), cell movement (motility) and controlled cell death (apoptosis).	[82]
miR-8066	NA	TGFb	Regulates various cell activities inside the cell, including the growth and division (proliferation) of cells, the maturation of cells to carry out specific functions (differentiation), cell movement (motility) and controlled cell death (apoptosis).	[82]

Legend: NA = not available.

## Data Availability

Data are contained within the article.
